# Complete Genome Sequence of Mycoplasma gallopavonis Type Strain WR1

**DOI:** 10.1128/MRA.01256-18

**Published:** 2018-11-29

**Authors:** Helmut Hotzel, Herbert Tomaso, Anne Busch

**Affiliations:** a Friedrich-Loeffler-Institut, Institute of Bacterial Infections and Zoonoses (IBIZ), Jena, Germany; University of Delaware

## Abstract

Here, we report the complete genome sequence of Mycoplasma gallopavonis type strain WR1 (= ATCC 33551 = NCTC 10186), which is a common microorganism in eastern wild turkeys and is considered a nonpathogenic commensal in turkeys.

## ANNOUNCEMENT

Mycoplasma is a genus within the class Mollicutes. Mycoplasma cells lack a cell wall around their cell membranes, and they are the smallest known self-replicating prokaryotes. They are small in genome and cell size and thus deficient in genes controlling biosynthetic pathways, and they are reliant on their host for the provision of many essential nutrients (1).

Mycoplasma gallopavonis was first described in 1982 as a Mycoplasma serovar of avian origin (2). M. gallopavonis can often be isolated from free-ranging eastern wild turkeys (Meleagris gallopavo silvestris), and it has not been considered a pathogenic avian Mycoplasma species (3, 4). To date, very little is known about the role of M. gallopavonis in poultry, such as whether the bacterium can act as a primary pathogen or an opportunistic secondary pathogen. The lack of published genome sequences has limited the molecular characterization of M. gallopavonis and the elucidation of its significance for animal health.

Mycoplasma gallopavonis type strain WR1, also known as ATCC 33551 and NCTC 10186, was received from a culture stock from Denmark in 1984. DNA for whole-genome sequencing was prepared from colonies grown on nutrient broth agar culture plates, using a High Pure PCR template preparation kit (Roche Diagnostics GmbH, Mannheim, Germany). The sequencing library was generated using the Nextera XT DNA library prep kit (Illumina, Inc., San Diego, CA). From an Illumina MiSeq run with an average read length of 300 bp and an expected insert size of 350 bp, 408,000 paired-end reads were generated, with a Phred score averaging >38 (mean sequencing depth of >200 reads, with a standard deviation of 60 bp). Further processing included quality trimming and assembly (included in SPAdes 3.12.1. in Bayes Hammer mode [–careful]) (5). Analysis of the data was performed with QUAST v4.3 and Bandage 0.8.1, using standard settings (6, 7). Filtering of the sample was performed by removing contigs with coverage less than 25× and a size below 850 bases. The assembly was finalized by a removal of contaminations with Kraken (8). The genome assembly was represented by 91 contigs with an *N*_50_ contig length of 15,045 bp, in which the largest contig had 39,296 bp. Annotation was performed with Prokka (9), using the code for Entomoplasmatales and Mycoplasmatales (10). Annotation features include 603 coding sequence(s) (CDS), 4 rRNAs, 31 tRNAs, and 1 transfer-messenger RNA (tmRNA), for a total sequence length of 795,497 bp. The GC content was calculated to be 28.14%. Analysis with Rapid Annotations using Subsystems Technology (RAST) and SEED (also in standard settings [9], using the code for Mycoplasmatales) (11, 12) revealed the closest neighbors, M. synoviae 53, M. mobile 163K, and M. pulmonis UAB CTIP, and thus might be important to differentiate mycoplasmas in a diagnostic context.

### Data availability.

The whole-genome sequence of Mycoplasma gallopavonis type strain WR1, also known as ATCC 33551 and NCTC 10186, was submitted under GenBank accession number QXGN00000000, BioProject accession number PRJNA488011, and BioSample accession number SAMN09935187. Primary data were deposited in the NCBI primary data archive, SRA, with the reference number SRP159222. The version described in this paper is the first version.
